# Size Variation in Flower Petals of Chinese Animal‐Pollinated Plants in Response to Climatic and Altitudinal Gradients

**DOI:** 10.1002/ece3.71396

**Published:** 2025-05-12

**Authors:** Siyu Chen, Jiayi Lu, Yuran Dong, Yao Li, Lingfeng Mao

**Affiliations:** ^1^ Co‐Innovation Center for Sustainable Forestry in Southern China, Laboratory of Biodiversity and Conservation College of Ecology and Environment, Nanjing Forestry University Nanjing China

**Keywords:** angiosperms, animal‐pollinated pants, China, ecological gradients, environmental adaptation, petal size, reproductive strategy

## Abstract

The evolutionary adaptations of plant reproductive structures, including angiosperm petal size, are driven by a combination of natural selection influenced by ecological conditions. While previous studies have emphasized pollinator‐driven selection on petal traits, significant gaps remain in understanding how abiotic factors, biotic interactions, and life‐history trade‐offs jointly shape petal size across broad environmental gradients. This study integrates macrogeographic analyses of 10,228 animal‐pollinated angiosperm species across China's diverse climatic regions, combining trait data from national flora databases, species distribution records, and high‐resolution climate variables. Using hierarchical regression, variance partitioning, and threshold detection models, we disentangle the effects of altitude, latitude, temperature, and precipitation on absolute petal size and its ratio to plant height (MR), while contrasting woody and herbaceous life histories. Key findings reveal: (1) nonlinear thresholds in environmental drivers, with herbaceous petal size declining sharply above 3200 m altitude and 1100 mm annual precipitation; (2) altitude as the dominant predictor of MR, explaining 30% of variance, particularly in alpine zones where floral conspicuousness increases despite plant dwarfing; (3) divergent strategies between woody and herbaceous species, where woody plants prioritize absolute petal size in warm climates, while herbaceous species amplify MR under high‐altitude stress; and (4) climate‐geography interactions explaining 62%–71% of trait variation, highlighting context‐dependent trade‐offs between pollinator attraction and stress tolerance. This work provides a comprehensive framework linking petal size traits to multivariate environmental gradients at continental scales, offering critical insights into plant adaptive strategies under climate change and emphasizing altitude‐mediated selection as a key driver of floral diversity.

## Graphical abstract

This study investigates the variation in flower petal size and the petal‐to‐plant height ratio among 10,228 animal ‐ pollinated angiosperm species across different climatic and altitudinal gradients in China. Results indicate that larger petal sizes are associated with higher temperatures and precipitation, while altitudinal variations significantly affect the petal‐to‐plant height ratio. These findings highlight the influence of environmental factors on floral morphology and underscore distinct geographical patterns in floral trait distribution.

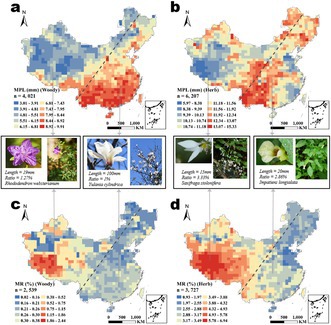

## 1 Introduction

In angiosperms, floral morphology is pivotal to reproductive success, with petal size being a fundamental functional trait implicated in diversification processes (Crane et al. 1995; Campbell and Powers 2015). Petals are typically regarded as the most visually prominent reproductive structures, and variations in their color, shape, and size directly affect pollinator visitation rates and behaviors (Irish 1998). While sepals—particularly petaloid sepals in some taxa (e.g., *Helleborus*)—can contribute to pollinator attraction through color mimicry (Landis et al. 2012; Roeder 2021), petals exhibit stronger macroclimatic responsiveness due to their direct role in thermoregulation and pollinator signaling (Kuppler and Kotowska 2021). However, fewer experiments have quantified petal differences and climate drives on a large scale.

While pollinator co‐evolution is frequently emphasized by ecologists (Johnson and Steiner 2000; Hegland and Totland 2005), it is important to recognize that multiple factors—including antagonistic biotic interactions, abiotic environmental elements, and life‐history trade‐offs—also exert significant influence on flower size (Galen 1999). Even environmental factors can be similar in intensity to pollination‐mediated selection on flower size (Caruso et al. 2019; Sugiyama and Bazzaz 1998). Additionally, the life‐history strategies embraced by plants, which integrate environmental, ecological, and evolutionary determinants, heavily impact petal size (Goodwillie et al. 2010). Consequently, discerning the interplay between petal size and environmental parameters facilitates a greater understanding of plant adaptive responses to variable conditions.

Climatic variations, primarily characterized by fluctuations in temperature and precipitation, manifest distinctly across geographical gradients (de Frenne et al. 2013). Petal size evolution, as hypothesized from co‐evolutionary frameworks, is closely associated with local pollinator adaptations to specific climatic contexts (Toji et al. 2021). In cool climates, larger petals may enhance thermoregulation and visibility to compensate for pollinator scarcity (Koski et al. 2024; Arroyo et al. 1985; Bingham and Orthner 1998), whereas excessive rainfall selects for smaller, rain‐resistant morphologies to prevent pollen washout (Lawson and Rands 2019). Larger petals can increase the visibility of the flower, making them more noticeable to pollinators from a distance (Paterno et al. 2020). Additionally, larger petals can also absorb more solar radiation (van der Kooi et al. 2019), which may create warmer microclimates that are more attractive to pollinators such as bees (Dyer et al. 2006). However, these adaptations are context‐dependent: in tropical regions with high florivory pressure, smaller petals may evolve to balance pollinator attraction and enemy escape (Boaventura et al. 2022).

Flower development demands significant biomass and nutritional investment into floral structures (Cruden and Lyon 1985; Méndez and Traveset 2003). Several studies have shown plants altering their resource allocation between reproduction and vegetative growth under drought stress (Arssen et al. 2014; Arssen 2015; Torices et al. 2018). Plant height, which correlates with flower size, also critically influences reproductive success (Tracey and Aarssen 2019). Taller plants with larger petals may be more attractive to pollinators (Schlinkert et al. 2016). As a result, plants can alter the allocation of resources to reproductive organs or nutritive organs (Raguso 2021; Caruso et al. 2019). Previous studies have shown that plants tend to become smaller as altitude increases, and the vegetative parts of plants also become smaller (such as leaf size), while the size of inflorescences does not change significantly(Körner 2021). This implies that the display of flowers will be more prominent. Thus, quantifying the ratio of petal size to plant height can yield insights into adaptive plant strategies.

A factor of petal sizes that is less discussed is the indirect effect of climate through natural enemies of flower petals. The ‘enemy‐escape hypothesis’ posits that plants may evolve towards less conspicuous flowers to balance the trade‐off between attractiveness to pollinators and susceptibility to florivores (Galen 1999). For instance, in hot, wet tropical regions where florivory rates are high, plants may develop smaller petals to mitigate this detrimental pressure (Boaventura et al. 2022).

We propose a hypothesis regarding petal size variation across different habitats. Specifically, we posit that angiosperms may develop distinct reproductive strategies in response to extreme environmental stress. One potential strategy involves increased reproductive investment, manifested through larger petal sizes or greater reproductive expenditure, aimed at maximizing reproductive success (Körner 2021). Alternatively, plants might either reduce reproductive investment, resulting in smaller petal sizes under harsher conditions (Teixido and Valladares 2014; Hou et al. 2022), or maintain stable petal sizes while adopting compensatory strategies, such as increasing flower production (Fabbro and Körner 2004).

To account for the functional relevance of pollination strategies, our study incorporates species‐level traits related to the mode of pollination. Approximately 90% of angiosperm species rely on animals for cross‐pollination, highlighting the critical role of biotic interactions in shaping floral traits and reproductive strategies (Ollerton et al. 2011; Tong et al. 2023). To refine our analysis, we leveraged existing databases, such as the Global Biodiversity Information Facility (GBIF), and filtered out abiotic pollination species. This ensures that our dataset emphasizes species whose petal sizes are more likely influenced by pollinator‐mediated selection.

Our study leverages a macrogeographic approach to demonstrate variation in petal size across different climatic regions within China—a country exhibiting a broad spectrum of global climate extremes (Piao et al. 2010; Zhang et al. 2017; Mohtadi et al. 2016). China serves as both a center of angiosperm diversification and a repository of its evolutionary history, making it an ideal setting for examining these relationships (Ren et al. 2018; Lu et al. 2018). Utilizing a comprehensive dataset comprised of 10,228 Chinese angiosperm species, we explore the spatial variation in petal size and analyze its associations with several climatic factors across diverse habitats, while also considering pollination strategies and other relevant traits. Our findings suggest that plants generally develop more conspicuous floral displays, encompassing both absolute flower size and the ratio of flower size to overall plant size, highlighting a potential strategy for reproductive assurance under varying environmental conditions.

## 2 Materials and Methods

### 2.1 Data Collection

We compiled petal size and the maximum length and width of each species' petal traits from the Flora of China (FOC, http://www.iplant.cn/foc/) and Flora Republicae Popularis Sinicae (FRPS, http://www.iplant.cn/frps). Our study focused on assessing the characteristics of individual flowers rather than whole inflorescences. Each flower was treated as a separate entity to ensure precise measurements of petal size and other relevant traits. If the study involved species known for clustered flowers, we specified that our measurements pertained to individual flowers within those clusters. We referenced the classification of corolla size (Wang et al. 2020) and categorized species as (1) species where petal size was explicitly recorded, from which we calculated the size using the length and width of petals; (2) species with only petal diameters recorded, from which we recorded the diameter as the length of the petal, and the width was equal to the length; (3) species with gamopetalous flowers, from which we recorded the length and width of the dehisced part, which was regarded as the display size of petals; (4) for Asteraceae, we recorded the tongue size as petal display size; (5) For species with the butterfly‐shaped corolla, we selected the largest recorded data among flag, wing, and keel. We removed several families where flower and inflorescence data were not well defined, as well as some families for which flower descriptions were not given, including the Poaceae, Dipsaceae, Lemnaceae, and Ruppiaceae (Goodwillie et al. 2010).

Referring to the study by Tong et al. (2023), we utilized the Global Biodiversity Information Facility (GBIF, https://www.gbif.org/) to examine the pollination modes of each species. We filtered out non‐biotic pollination species, retaining only those that exhibit biotic and ambophilous pollination strategies. In all, we were able to compile petal data from 10,228 species of 1,283 genera in 169 families. Of these species, 39.31% (4, 021 species) were classified as woody and 60.69% (6, 207 species) were herbaceous. We also recorded the height of the total plant from the Flora of China (FOC, http://www.iplant.cn/foc/) and Flora Republicae Popularis Sinicae (FRPS, http://www.iplant.cn/frps). Then, we calculated the ratio of petal length to plant height, converting it to apercentage.

We used species distribution data from Lu et al. (2018), which contained 1,409,239 distribution records of 26,978 Chinese angiosperms. In order to illustrate the variation of petal size variables across space, we utilized a map averaged into 100‐km grid cells (Lu et al. 2018). We obtained climate data from the WorldClim database Version 2.1 (http://www.worldclim.org/) with a spatial resolution of 10 min (Fick and Hijmans 2017), and used ArcMap10.2 to match the climate data and calculate the average in each grid.

### 2.2 Data Analysis

**FIGURE 1:**
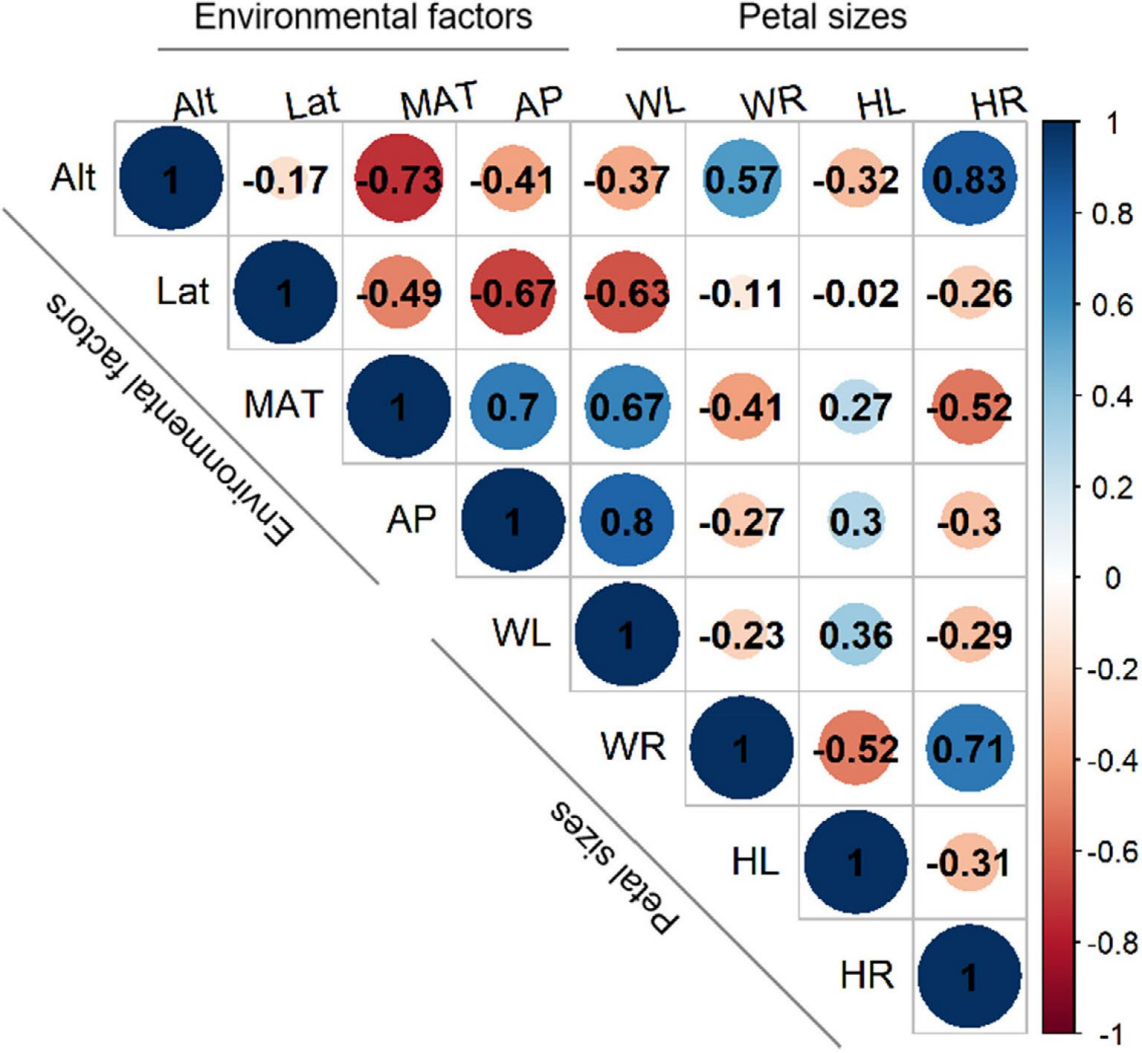
Pearson correlation matrix of petal sizes and environmental variables. This correlation matrix illustrates the relationships between the independent variables (Altitude, Latitude, Mean Annual Temperature, and Annual Precipitation) and the dependent variables (Mean petal length of woody species, Mean ratio of petal length to plant height of woody species, Mean petal length of herbaceous species, and Mean ratio of petal length to plant height of herbaceous species). The values represent Pearson correlation coefficients (*r*), with a range from −1 (perfect negative correlation) to +1 (perfect positive correlation). The significance of the correlations can be assessed based on the associated *p*‐values. All correlations are statistically significant (*p* < 0.05) except for the relationship between Herbaceous mean petal length (HL) and Latitude (Lat) (*p* = 0.535). Alt, altitude; AP, annual precipitation; HL, herbaceous mean petal Length; Lat, latitude; MAT, mean annual temperature; WL, woody mean petal length; WR, herbaceous mean ratio of petal length to plant height; WR, woody mean ratio of petal length to plant height.

The petal size (MPL) and the mean ratio of petal length to plant height (MR) as response variables were analyzed, with altitude, latitude, annual precipitation (AP), and mean annual temperature (MAT) as independent variables. Prior to model construction, we assessed multicollinearity among all predictors (altitude, latitude, MAT, AP) using Pearson correlation coefficients (Figure 1). Strong collinearity was observed between altitude–MAT (*r* = −0.73) and MAT–AP (*r* = 0.7, Dormann et al. 2013). However, we retained these variables because: (1) They represent distinct ecological mechanisms, which MAT represents thermal energy availability, AP reflects water supply, and altitude integrates abiotic stressors (e.g., hypoxia, UV‐B); (2) Statistical validation: Variance partitioning and hierarchical analysis isolated their independent contributions; (3)Threshold divergence: MAT and AP exhibited non‐overlapping critical points in segmented regressions, suggesting independent triggers (Dormann et al. 2013).

We initially quantified pairwise relationships between petal traits and individual environmental variables using linear models. For non‐linear responses (e.g., latitude, precipitation), quadratic terms were incorporated, and segmented regression models were fitted using the segmented package in R (Muggeo 2008) to identify critical thresholds in trait‐environment relationships.

To disentangle joint and independent effects of climate (MAT, AP) versus geography (elevation, latitude), we performed redundancy analysis (RDA)‐based variation partitioning (vegan package; Dixon 2003). This method quantified variance explained by: pure climate effects (independent of geography), pure geographic effects (independent of climate), and shared variance (climate‐geography interactions).

Generalized linear models (GLM, Gaussian family) were used for statistical analysis in this study. We estimated the linear relationship between relevant variables and response variables. It was performed using the glm function in R. The MPL and MR as response variables were analyzed with altitude, latitude, annual precipitation (AP), and mean annual temperature (MAT) as independent variables, adding their interactions. Then, based on the certain weighting of each factor and the stepAIC function, the optimum selection models of the multiple regression models were built. To estimate the relative importance of each explanatory variable, we used a hierarchical partitioning in each glm model, using the “rdacca.hp” package in R (Lai et al. 2022).

## 3 Results

### 3.1 Spatial Patterns of Petal Size

**FIGURE 2:**
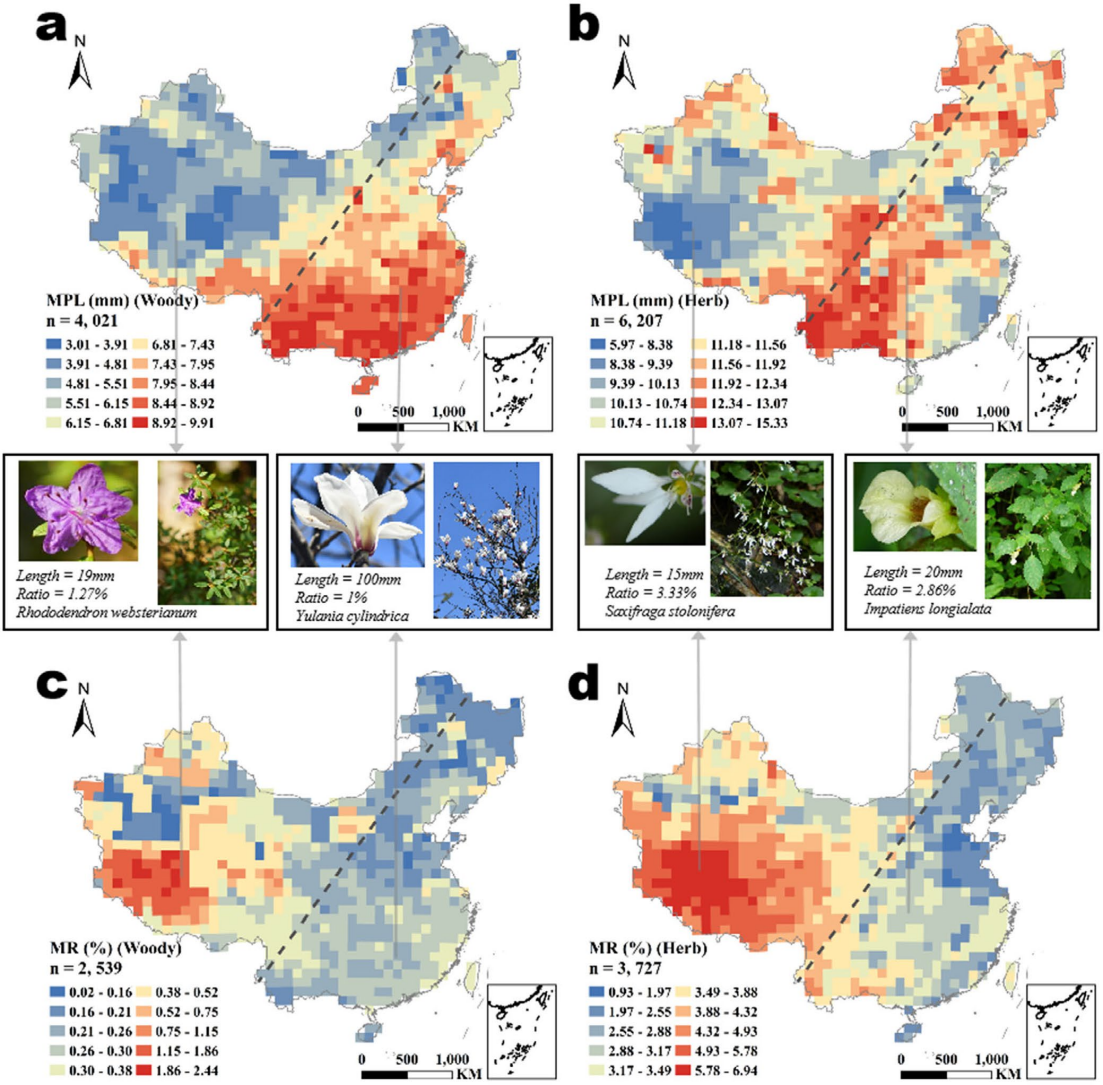
Spatial patterns of petal sizes in China. Petal sizes pattern, shown as the mean petal length (MPL, mm) for woody species (a) and herbaceous species (b). The pattern of petal relative size, represented as the mean ratio of petal length to plant height (MR, %) in each grid for woody species (c) and herbaceous species (d). The dashed line indicates the Hu Line demarcating eastern and western China.

The spatial heterogeneity of petal size across China reflects distinct adaptive strategies between woody and herbaceous plants (Figure 2). Woody species exhibited maximal petal sizes in the warm, humid south, whereas herbaceous plants peaked in the climatically stable southwest. Notably, both groups converged towards minimal petal sizes in the arid northwest and the Qinghai‐Tibet Plateau, suggesting that extreme cold and aridity impose universal constraints on floral investment.

### 3.2 Environmental Correlates of Petal Size

**FIGURE 3:**
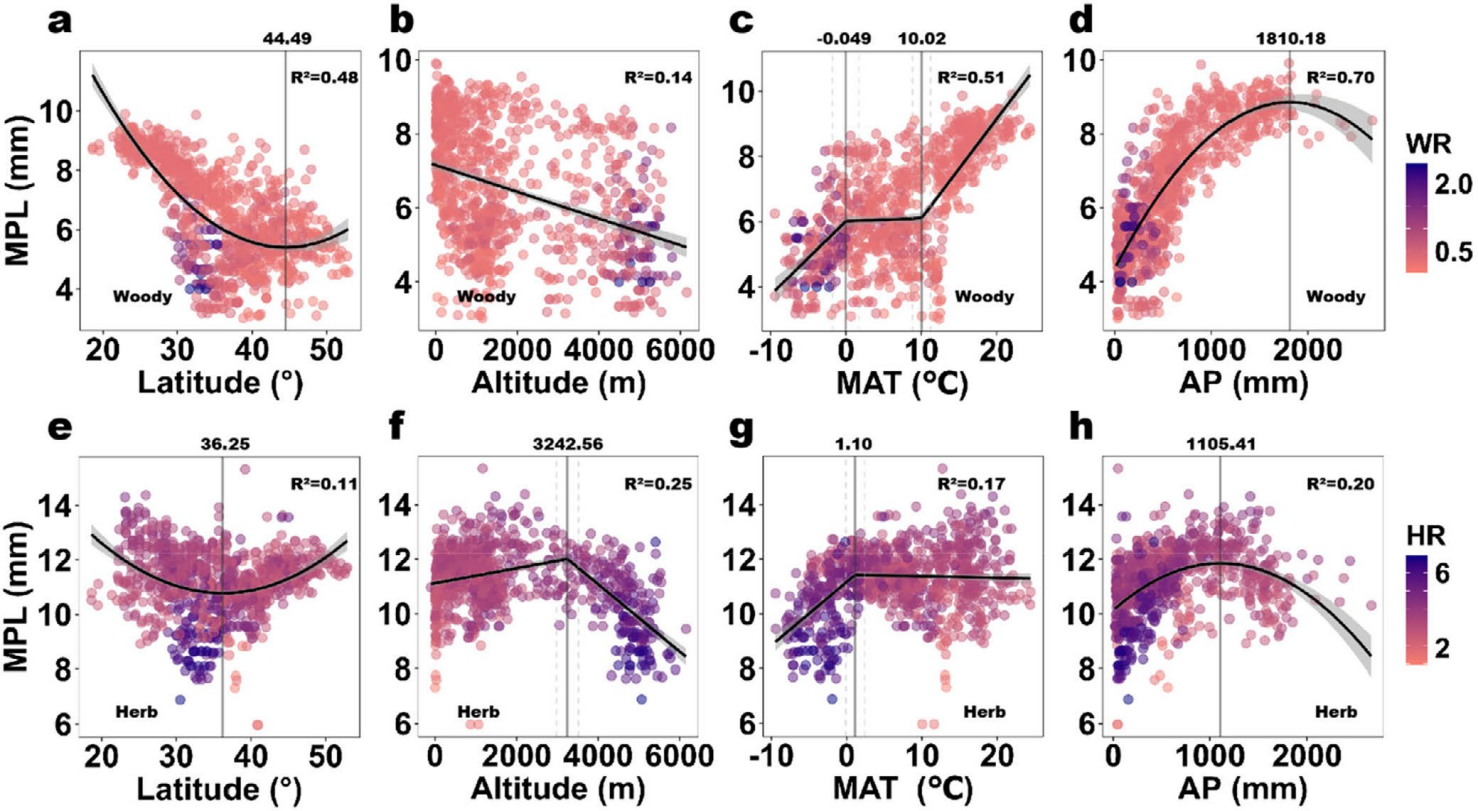
Variation trends of mean petal size (MPL) in relation to environmental factors. Quadratic Regression and Segmented Regression (linear model, LM) of mean petal length (MPL) change trend with changing environmental factors for both woody species (upper half of this figure, a, b, c, d) and herbaceous species (bottom half of this figure, e, f, g, h). Vertical lines (gray) in quadratic regressions represent the axes of corresponding functions and are also the extreme points (a, d, e, h). Vertical lines (gray) in segmented regressions represent the breakpoints, and the dotted lines represent ± SE (dotted lines) confidence interval (c, f, g). The color of the scatter points indicates the mean ratio of petal length to plant height (MR) of the corresponding species type.

Our analysis confirms that petal size increases towards the poles, as it correlates quadratically with latitude (Figure 3a for woody species, Figure 3e for herbaceous species). Their minimum values both occur in the mid‐latitude range (between 30° and 60°, Figure 3a,e). For altitude, petal size generally exhibited a negative correlation in both woody and herbaceous species (Figure 3b,f). However, herbaceous species displayed a distinct breaking point at approximately 3200 m, beyond which petal size decreased sharply (Figure 3f). This altitude corresponds to the transition to alpine ecosystems, suggesting that herbaceous plants may be more sensitive to high‐altitude environmental stresses. Below this threshold, a slight initial increase in herbaceous petal size was observed (with a low slope), possibly reflecting marginally optimal conditions for floral development at moderate elevations.

When fitting petal sizes to variation in temperature and precipitation, we found the following patterns (Figure 3c,d for woody species, Figure 3g,h for herbaceous species): For mean annual temperature (hereafter referred to as temperature), petal size increased rapidly from low temperatures up to approximately 0°C for both woody and herbaceous plants. Beyond this threshold, herbaceous petal size appeared to reach an asymptote (Figure 3g). In contrast, woody plants exhibited a more variable pattern with multiple breaking points—woody petal size leveled off at intermediate temperatures (between 0°C and 10°C), and then increased rapidly again with rising temperatures above 10°C (Figure 3c).

Petal size also correlated quadratically with annual precipitation for both groups (Figure 3d,h). For woody plants, petal size increased with precipitation up to a threshold of approximately 1800 mm, beyond which it began to decline (Figure 3d). In contrast, herbaceous plants showed a similar trend but with a lower threshold of around 1100 mm (Figure 3h). These findings suggest that while both groups respond to precipitation, their optimal ranges differ, likely due to life‐history strategies and resource allocation patterns.

### 3.3 Environmental Correlates of Petal Relative Size

Petal relative size (MR, mean ratio of petal size to plant height) exhibited consistent negative correlations with temperature, precipitation, and altitude across both woody and herbaceous species (Figure 1). Variance decomposition and hierarchical partitioning analyses identified altitude as the most significant determinant of MR, independently explaining approximately 30% of the variation in both groups (Table 1).

**TABLE 1:** Individual importance (%) of the environmental and geography variables add interactions to explain mean petal length (MPL) and mean ratio of petal size to plant height (MR), and importance based on the variable type.

Variables	Unique	Average. share	Individual	I. perc (%)	*p*
M1: Woody‐MPL model (GLM). *R* ^2^m = 0.7528***					
AP	0.0197	0.1757	0.1954	25.96	***
Alt	0.0166	0.0421	0.0587	7.80	***
Lat	0.0567	0.0887	0.1454	19.31	***
Alt*AP	0.0021	0.0313	0.0334	4.44	**
Lat*AP	0.0347	0.1533	0.1880	24.97	***
Alt*MAT	0.0072	0.0434	0.0506	6.72	***
Lat*MAT	0.0071	0.0742	0.0813	10.80	***
M2: Herb‐MPL model (GLM). *R* ^2^m = 0.4661***					
AP	0.0127	0.0217	0.0344	7.38	***
MAT	0.0155	0.0154	0.0309	6.63	***
Alt	0.0301	0.0440	0.0741	15.90	***
Alt*AP	0.0407	0.0336	0.0743	15.94	***
Lat*AP	0.0112	0.0390	0.0502	10.77	***
Alt*MAT	0.0342	0.1316	0.1658	35.57	***
Lat*MAT	0.0343	0.0021	0.0364	7.81	***
M3: Woody‐MR model (GLM). *R* ^2^m = 0.4545***					
AP	0.0097	0.0229	0.0326	7.17	***
MAT	0.0070	0.0467	0.0537	11.82	***
Alt	0.0305	0.1165	0.1470	32.34	***
Lat	0.0027	0.0465	0.0492	10.83	*
Alt*AP	0.0712	−0.0193	0.0519	11.42	***
Alt*MAT	0.0023	0.0640	0.0663	14.59	*
Lat*MAT	0.0074	0.0464	0.0538	11.84	***
M4: Herb‐MR model (GLM). *R* ^2^m = 0.7340***					
AP	0.0081	0.0401	0.0482	6.57	***
MAT	0.0054	0.0751	0.0805	10.97	***
Alt	0.0185	0.2305	0.2490	33.92	***
Lat	0.0028	0.0853	0.0881	12.00	**
Alt*AP	0.0149	0.0487	0.0636	8.66	***
Lat*AP	0.0080	0.0616	0.0696	9.48	***
Alt*MAT	0.0041	0.0434	0.0475	6.47	***

*Note:* *p < 0.05, **p < 0.01, ***p < 0.001. All models are the best subset selection.

Abbreviations: Alt, altitude; AP, annual precipitation; Lat, latitude; MAT, mean annual temperature.

**FIGURE 4:**
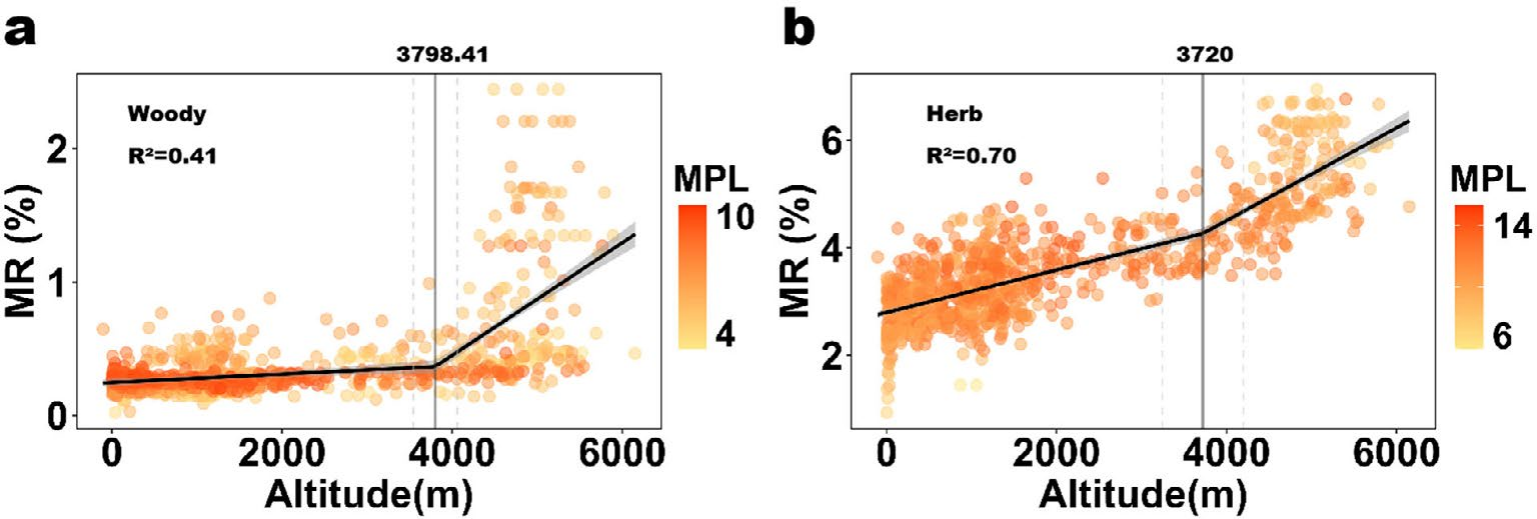
Variation trends of mean ratio of petal size to plant height (MR) with altitudinal gradient. Segmented Regression (LM) of the mean ratio of petal size to plant height (MR) along an altitudinal gradient for both woody species (a) and herbaceous species (b). Vertical lines represent the breakpoints, and the dotted lines are the standard‐error confidence intervals. The color of the scatter points indicates the mean petal length (MPL) of the corresponding species type.

A critical elevation range between 3700 and 3800 m was observed, beyond which the relationship between MR and altitude became significantly steeper (Figure 4a,b). This range corresponds to the Qinghai‐Tibet Plateau, where extreme environmental conditions likely drive stronger selection pressures on floral traits. Notably, the relationship was more pronounced in woody species than in herbaceous plants, suggesting differential adaptive responses to high‐altitude stresses.

### 3.4 Joint Effects of Climate and Geography

**FIGURE 5:**
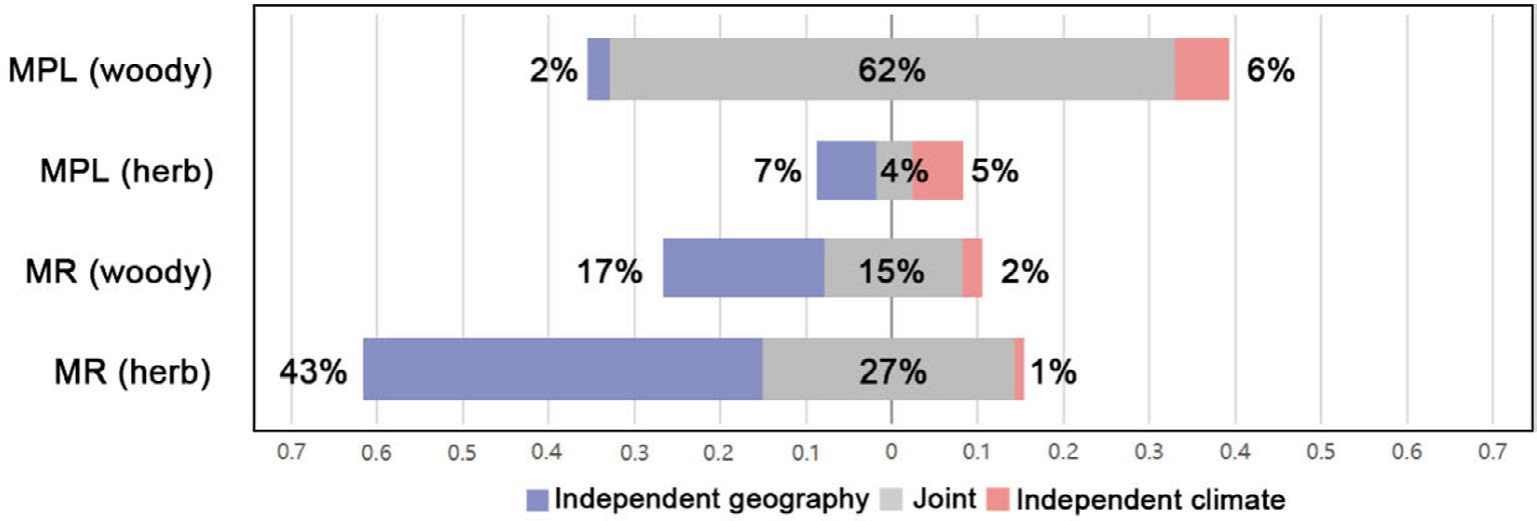
Climatic and geographical factors explain the two‐dimensional spectrum of petal size variation. Percentage variation is explained by geography (violet, percentages on the left), climate (peach, percentages on the right), and jointly (grey, percentages in the middle) for each petal size group. Total bar length = total *R* ^2^ explained by climate and geography. The joint effect is the fraction explained by both climate and geography together and is split equally among them. The independent effect is the fraction of *R* ^2^ explained exclusively by either soil or climate variables.

To disentangle the effects of climate and geography, we assessed their joint and independent contributions to petal size and MR variability (Figure 5). For petal size, environmental factors explained a greater proportion of variance in woody plants: climate and geography jointly accounted for 62% of the variance, with independent climate and geography effects contributing an additional 6% and 2%, respectively, totaling 70% of the variance explained.

In contrast, for petal relative size, herbaceous plants showed higher explainability: climate and geography jointly explained 27% of the variance, with independent climate and geography effects contributing an additional 1% and 43%, respectively, totaling 71% of the variance explained. These results highlight that while woody petal size is more strongly influenced by the combined effects of climate and geography, herbaceous petal relative size is predominantly shaped by geographic factors, particularly altitude.

## 4 Discussion

Our comprehensive analysis reveals that petal size exhibits notable variability among animal‐pollinated angiosperms spanning the diverse environmental and biogeographic gradients of China. This variation may emerge from a three‐way interplay between abiotic constraints, biotic interactions, and developmental trade‐offs, with altitude emerging as the dominant driver of floral trait variation.

Contrary to prior assumptions, our study found that colder climates tend to result in smaller flowers. This may be attributed to the inhibitory impacts of low temperatures on plant growth and the ability of plants to allocate resources towards floral development (Guo et al. 2010). This phenomenon may arise as a consequence of plastic responses to reduced resource availability or as a result of adaptive evolution (Gibbin et al. 2017). Higher temperatures also provide more absorbable heat for the petals. Larger petals contribute to the accumulation and retention of heat, leading to increased warmth in the flower, which in turn enhances pollinator visitation (van der Kooi et al. 2019). This thermoregulatory advantage may be particularly critical for animal‐pollinated species in cooler environments.

Likewise, the availability of water is crucial for the growth and development of plants (Gupta et al. 2020; La Pierre et al. 2011), and it can significantly impact the allocation of resources for plant reproduction (Gupta et al. 2020; Caruso et al. 2019). Certainly, there is a strong correlation between water stress and flower size, as evidenced by a reduction in both the width and length of petals in response to escalating drought conditions (Kuppler and Kotowska 2021). Additionally, excessive rainfall may reduce pollinator activity (Antiqueira et al. 2020), potentially affecting selection for large petals in these environments. However, our refined analysis identified a threshold, particularly evident in herbaceous plants, suggesting that beyond a certain precipitation level, flower size decreased. One possible explanation for this phenomenon could be that in regions with high levels of precipitation, smaller herbaceous petals serve to minimize the surface area in contact with the rain, thereby helping to preserve the internal structure of the flower (Lawson and Rands 2019).

Temperature and precipitation also limit the distribution and activity of pollinators (Elle and Carney 2003). Similarly, lower latitudes and altitudes exhibit greater abundance and diversity of pollinators (Ollerton 2017). In such environments, there is competition among plants for pollinators (Ogilvie et al. 2017), and those with larger petals and a more conspicuous floral display may have a competitive advantage (Midolo and Wellstein 2020; Paterno et al. 2020). Simultaneously, larger petals are also vulnerable to herbivores and folivores (Teixido et al. 2016). Consequently, in the presence of abundant resources, plants exhibit trade‐offs in petal size, balancing floral attraction for pollinators and consumers. Plants that develop and maintain large petals incur an enormous resource allocation cost (Teixido and Valladares 2014). Nevertheless, plants in harsh environments also exhibit a tendency for increased petal relative size. The findings of our study suggest that petal relative size, and by extension, the allocation of resources to reproduction, is strongly influenced by altitude and the climate factors that covary with altitude, such as temperature and precipitation (Körner 2007). Additionally, biotic factors such as the diversity and abundance of pollinators and herbivores also play a substantial role in shaping these patterns (Benzina et al. 2020).

Elevation dominates petal relative size variation as a composite selector integrating stressful environmental conditions. At high altitudes, the selective pressure for conspicuous flowers is pronounced due to limited pollinator availability for animal‐pollinated species. On one hand, plants exhibit a tendency to be more conservative with resources in response to increasingly severe abiotic stress (Midolo and Wellstein 2020). Plants at higher altitudes typically exhibit reduced size (Guo et al. 2010; Mao et al. 2020) and increased reproductive costs (Chapurlat et al. 2015). Their diminutive size results in the reproductive organs, such as petals, being relatively prominent. On the other hand, plants need to trade off vegetative growth and reproduction. As elevation increases, plants exhibit a reduction in height (Mao et al.), the ratio of petal size to plant height demonstrates an increase, suggesting a more pronounced petal appearance. This “giant flower” phenomenon in alpine flowering plants aligns with strategies to enhance pollinator attraction under low‐density pollinator communities (Körner 2021). In high‐altitude environments characterized by a scarcity of pollinators, conspicuous flowers are more readily discernible to pollinating agents. Simultaneously, this could confer advantages to plants in high‐altitude environments, where pollinators tend to exhibit greater specialization (Berrached et al. 2017). This provides a selective advantage to plants that produce flowers that are more readily detected by pollinators (Johnson et al. 1995). The increased allocation of resources to reproductive organs in response to elevation is an adaptive mechanism that enhances sexual reproduction in challenging alpine environments (Fabbro and Körner 2004), such as the Qinghai‐Tibet Plateau (Sun et al. 2014).

Although the responses of woody and herbaceous petals to environmental variation were similar, there were some distinct differences. Specifically, we found that the petal size of woody plants was more sensitive to environmental variation than herbaceous plants, while herbaceous plants' reproductive strategies are more strongly tied to the environment than woody plants. One reason for this is that woody species tend to be much larger than herbaceous species (Petit and Hampe 2006). Large plant size also increases reproductive success. On a large geographical scale, the petal sizes of woody plants vary along environmental gradients. For example, woody species grow very slowly at low temperatures and high altitudes (> ~3400 m, Amici et al. 2013; Rossi et al. 2007), and they have larger petal displays to ensure reproductive success. Different from most woody plants, herbaceous plants usually tend to have shorter life cycles. The survival and reproduction of herbaceous plants are more susceptible to external environmental factors (Compagnoni et al. 2021). They need to respond to changes in the environment, such as rapidly increasing the relative size of petals in harsh environments. Large‐scale environmental differences have more influence on the potential resource allocation of herbaceous plants (Spicer et al. 2022). Microenvironmental factors within herbaceous communities, such as competition from neighboring plants or localized nutrient availability, may further exacerbate this sensitivity and influence floral trait evolution (Basnett et al. 2021).

While our study investigated large‐scale environmental gradients affecting petal size, it is important to acknowledge the role of microenvironments within plant communities, particularly for herbaceous plants. Microenvironments, such as differences in shading, soil moisture, and local wind patterns, can create substantial variation in resource availability and stress levels within a small spatial scale (Craine and Dybzinski 2013). These localized conditions may lead to different trade‐offs in resource allocation, especially for short‐lived herbaceous species, which are often more sensitive to external environmental fluctuations (Violle et al. 2009). For instance, herbaceous plants in shaded understory microhabitats may exhibit smaller petal sizes compared to plants in open, sunlit habitats due to reduced light availability and competition for resources (Lim et al. 2025). Although microenvironmental variation was not explicitly accounted for in our dataset, future studies should consider incorporating fine‐scale environmental data to better understand how these localized factors modulate petal size variation and resource allocation strategies. Despite the underlying patterns in plant reproductive strategies that we observed, we emphasize that floral size is highly variable and diverse within each grid cell, and our analyses were focused on mean effects, not their variance. By restricting our analysis to animal‐pollinated species, we reduce confounding effects from wind or cleistogamy systems but acknowledge that unmeasured traits (e.g., inflorescence architecture, floral longevity) may further mediate these relationships (Iwata et al. 2012; Wang et al. 2020).

## 5 Conclusion

In conclusion, our study demonstrates that petal size in animal‐pollinated angiosperms varies significantly across environmental gradients in China, driven by a complex interplay of abiotic constraints, biotic interactions, and developmental trade‐offs. The identification of threshold responses to temperature and precipitation highlights the nonlinear nature of these relationships, while the pronounced effects of elevation underscore the importance of stressful environments in shaping floral traits. These findings not only advance our understanding of plant reproductive strategies but also provide a framework for predicting how floral traits may respond to ongoing climate change. Future studies incorporating fine‐scale environmental data and unmeasured traits, such as floral longevity and nectar production, will further elucidate the mechanisms underlying these patterns.

## Author Contributions

**Siyu Chen:** conceptualization (equal), data curation (lead), formal analysis (lead), funding acquisition (equal), investigation (equal), methodology (lead), project administration (lead), resources (equal), software (lead), supervision (equal), validation (equal), visualization (lead), writing – original draft (lead), writing – review and editing (lead). **Jiayi Lu:** data curation (equal), formal analysis (equal), methodology (equal), software (equal), validation (equal), visualization (supporting), writing – original draft (supporting), writing – review and editing (supporting). **Lingfeng Mao:** conceptualization (lead), data curation (equal), formal analysis (equal), funding acquisition (lead), investigation (equal), methodology (lead), project administration (equal), resources (lead), software (equal), supervision (equal), validation (equal), visualization (equal), writing – original draft (equal), writing – review and editing (equal). **Yuran Dong:** data curation (supporting), formal analysis (supporting), investigation (equal), methodology (equal), software (equal), writing – original draft (equal), writing – review and editing (supporting). **Yao Li:** data curation (equal), formal analysis (equal), methodology (equal), software (equal), visualization (supporting), writing – original draft (supporting).

## Conflicts of Interest

The authors declare no conflicts of interest.

## Supporting information


Appendix S1.

## Data Availability

The datasets that support the findings of this study on the variation in flower petal size among Chinese animal ‐ pollinated angiosperms are available in the Supporting Information accompanying this manuscript.
